# Voluntary Vaccination through Self-organizing Behaviors on Locally-mixed Social Networks

**DOI:** 10.1038/s41598-017-02967-8

**Published:** 2017-06-01

**Authors:** Benyun Shi, Hongjun Qiu, Wenfang Niu, Yizhi Ren, Hong Ding, Dan Chen

**Affiliations:** 1 School of Cyberspace, Hangzhou Dianzi University, Hangzhou, 310018 China; 2 Key Laboratory of Complex Systems Modeling and Simulation, Ministry of Education, Hangzhou, 310018 China; 3 School of Computer, Wuhan University, Wuhan, 430072 China

## Abstract

Voluntary vaccination reflects how individuals weigh the risk of infection and the cost of vaccination against the spread of vaccine-preventable diseases, such as smallpox and measles. In a homogeneously mixing population, the infection risk of an individual depends largely on the proportion of vaccinated individuals due to the effects of herd immunity. While in a structured population, the infection risk can also be affected by the structure of individuals’ social network. In this paper, we focus on studying individuals’ self-organizing behaviors under the circumstance of voluntary vaccination in different types of social networks. Specifically, we assume that each individual together with his/her neighbors forms a local well-mixed environment, where individuals meet equally often as long as they have a common neighbor. We carry out simulations on four types of locally-mixed social networks to investigate the network effects on voluntary vaccination. Furthermore, we also evaluate individuals’ vaccinating decisions through interacting with their “neighbors of neighbors”. The results and findings of this paper provide a new perspective for vaccination policy-making by taking into consideration human responses in complex social networks.

## Introduction

Voluntary vaccination plays an essential role in achieving widespread immunity to many infectious diseases, such as smallpox and measles^1–3^. Epidemiological models suggests that there exists an interplay between human vaccinating behavior and the spread of infectious diseases^4–7^. Specifically, the vaccinating behavior of individuals depends mainly on how they weigh the risk of infection against the cost of vaccine complications, including the financial cost^8, 9^ side effects^10–12^, and so on. As the vaccine coverage increases, the unvaccinated individuals are more unlikely to become infected due to the effects of herd immunity^13–15^. On the other hand, if all individuals are aware of the way to exploit herd immunity, one would most likely act as a free rider on the vaccination of others. This paradox leads to a public-goods dilemma^16^ for disease eradication. In this case, to help design effective vaccinating policies, it would be essential to understand the interplay between disease prevalence, vaccine coverage and the dynamics of individuals’ vaccinating behaviors. In this paper, we focus mainly on investigating the dynamics of voluntary vaccination through individuals’ self-organizing behaviors in the face of a vaccine-preventable disease like smallpox and measles.

Great efforts have been made to reproduce the decision-making process of taking vaccination based on the game-theoretical analysis dovetailed with epidemic models^17–23^. For example, Bauch *et al*. have presented a Nash equilibrium on the vaccine coverage based on a vaccination game^17^. They have revealed that there is a clash between individuals’ self-interest and group interest with respect to smallpox vaccination, which suggests that voluntary vaccination fails to reach the level of vaccine coverage that is best the whole population^18^. Further, Bauch and Reluga *et al*. have studied the oscillations in vaccine uptake by assuming that individuals are rational and have complete knowledge about the population^24, 25^. They found that oscillations of vaccine uptake are more likely due to individual’ imitation behaviors and sensitivity to disease prevalence. To date, many studies have shown that game-theoretical analysis can well explain certain vaccination phenomena in a homogeneously mixing population^25–27^. However, in most cases, individuals are contact-structured in the form of social networks^28, 29^. Moreover, it is almost impossible for any individual to have complete knowledge about network structure of the whole population, which make it difficult to predict the dynamics of disease epidemics^30–33^, as well as human behavioral responses^34–36^.

Different from earlier compartmental models^6^, simulation models have been widely adopted to characterize complex interplay between individuals’ behavioral responses and the epidemic spreading on social contact networks^37–43^. Usually, individuals are assumed to act in their own interests and make vaccinating decisions based on their perceived risk of infection and vaccination in their neighboring environment. For example, Perisic and Bauch have shown that social contact structure can enable eradication of an infectious disease under voluntary vaccination^37, 38^. In their simulation, individuals decide to vaccinate when the perceived payoff of vaccinating is larger than that of not vaccinating during the epidemic process. Further, researchers have investigated several evolutionary strategies for individuals to adjust their vaccinating behaviors through interacting with their neighbors, such as imitation^40–43^, pairwise comparison^44^, birth-death and death-birth strategies^45^. Most these studies focus mainly on *pairwise interactions* between neighboring individuals, where *pure vaccinating strategy* is adopted to decide whether or not to vaccinate based on social learning and individuals’ past experiences in an evolutionary process.

In reality, people play different roles and contact with each other in groups. For example, family members live together; classmates attend classes together; colleagues have meetings together. Along this line, many studies have focused on modeling the epidemic dynamics and immunization on metapopulation networks^46, 47^ and multiplex networks^48–51^. Motivated by this consideration, in this paper, we formulate a social contact network as a “network of networks”^52–54^ by assuming that each individual together with his/her neighbors forms a locally-mixed group. Individuals within a group are homogeneously mixing and have complete information about other individuals’ vaccinating strategies. In this way, each individual may belong to several groups through network links with his/her neighbors. Such an assumption extends the pairwise interaction among individuals in most existing studies, and at the same time reflects the limitations of human knowledge and communication through the network structure. To the best of our knowledge, few studies have focused on the dynamics of voluntary vaccination in such networks^55^.

Build upon the above considerations and assumptions, here we aim to investigate the dynamics of individuals’ self-organizing vaccinating behaviors on different types of social contact networks. Following the existing studies of Bauch *et al*.^17, 18, 24, 25^, we adopt the Susceptible-Infected-Recovered (SIR) model to simulate the transmission of a vaccine-preventable disease like smallpox or measles. Specifically, we captures the strategic interactions of individuals in the following way. First, individuals randomly determine their initial probabilities of vaccination at the beginning of an evolutionary process. It should be noted that individuals adopt a mixed vaccinating strategy rather than a pure strategy, which can reflect their willingness to vaccinate. Then, individuals predict the risks of infection in their locally-mixed environments based on their neighbors’ strategies using an epidemiological model. Based on the perceived risks of infection, they further update their probabilities of vaccination to balance the costs of infection and vaccination. Finally, it is expected that such self-organizing behaviors among interconnected individuals would lead to a steady state about their willingness to vaccinate under voluntary vaccination. By doing so, our model offers a new perspective for analyzing individuals voluntary vaccinating behaviors, which does not rely on social learning among neighboring individuals, nor individuals’ past experiences about vaccination and infection from an infectious disease.

## Results

In this section, simulations are carried out to study individuals’ self-organizing vaccinating behaviors on three types of locally-mixed complex networks, they are: random regular networks, small-world networks, and scale-free networks. Specifically, the effects of infection force *β*, network structure and average degree *k* on the dynamics of voluntary vaccination are evaluated. Moreover, two scenarios of individuals’ decision-making process (i.e., with or without considering “neighbors of neighbors”) are simulated to investigate the relationships between individual degree and vaccinating strategy.

### The effects of infection force

**Figure 1:**
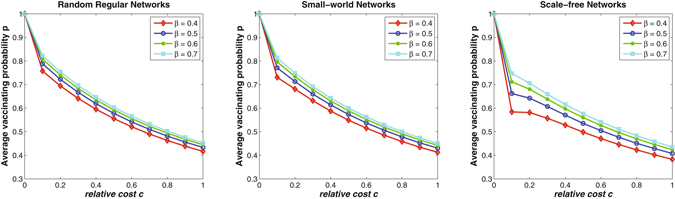
The effects of infection force on random regular networks, small-world networks, and scale-free networks. Each line shows the trend of average vaccinating probability of all individuals *p* as the relative cost *c* increases. While for each types of networks, the average degree of each network is set to be *k* = 4, and *p* is averaged over 50 independent network simulations.

The infection force (i.e., *β* in the SIR model) represents the number of effective contacts per susceptible individual per day that are sufficient to spread a disease (see Methods section for the description of the SIR model and its associated parameters). Therefore, it is natural and meaningful to study its effects on individuals’ vaccinating behaviors. Figure 1 shows the trends of average vaccinating probability of all individuals *p* as the relative cost *c* increases from 0 to 1. Without loss of generality, the value of *γ* is set to be 1/3 day^−1^ through all simulations. While the value of *β* is set to be 0.4, 0.5, 0.6, and 0.7, respectively. We have verified that such settings correspond to the *basic reproduction number R*_0_ in the range from 1.5 to 4 for all the three types of networks with average degree *k* = 4 and network size *N* = 5000. For each type of networks, the average vaccinating probability *p* are calculated from 50 independent network simulations. It can be observed that the average vaccinating probability *p* on all the three types of networks decreases when (i) the relative cost *c* increases, and (ii) the infection force *β* decreases. This is intuitive and reasonable because rational individuals will weigh the costs of vaccination and infection during their decision-making. A relative high cost *c* will decline their willingness to take vaccination (see Equation (11) in Methods section). Similarly, a small value of infection force *β* will relatively decrease individuals’ risks of infection (see Equation (10) in Methods section), and further decline their willingness to take vaccination. Since the effect of infection force *β* on disease epidemics and human vaccinating behaviors varies on different networks, therefore, it would be necessary to identify the pure effects of network structure.

### The effects of network structure

**Figure 2:**
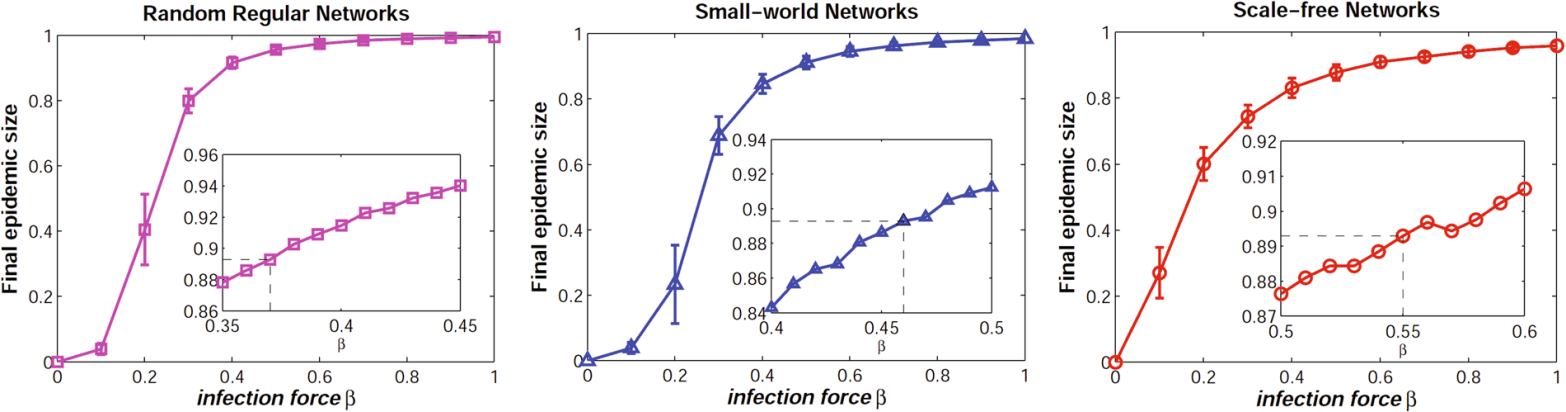
The final epidemic size with different values of infection force *β* on random regular networks, small-world networks, and scale-free networks. The results are averaged over 50 independent network simulations with size *N* = 5000 with average degree *k* = 4. The infection force *β* is chosen to make sure that the final epidemic size on each type of networks is the same as that of the well-mixed population with *R*_0_ = 2.5 and *γ* = 1/3. The insets show that the final epidemic size is around 0.893 when *β* = 0.37 for random regular networks, *β* = 0.46 for small-world networks, and *β* = 0.55 for scale-free networks.

To identify the pure effects of network structure, the infection force *β* is calibrated to make sure that the final epidemic size on each type of networks is the same as that of the well-mixed population without vaccination. By setting *R*_0_ = 2.5 and *γ* = 1/3 as a base case, simulation results in a well-mixed population show that the final epidemic size is around 0.893^40^. Accordingly, for each type of complex networks, the dynamics of epidemic process is simulated by testing various values of infection force *β*, while fixing the recovery rate *γ* = 1/3. Figure 2 shows the simulation results on the three types of networks with size *N* = 5000 and average degree *k* = 4, where the results are averaged over 50 independent network simulations. It can be observed that the final epidemic size in scale-free networks appears to be smaller than other types of networks for higher value of *β* but larger when *β* get lower enough. The results are consistent with previous findings^56^. According to the base case in a well-mixed population, it can be estimated that the final epidemic size is around 0.893 when *β* = 0.37 for random regular networks, *β* = 0.46 for small-world networks, and *β* = 0.55 for scale-free networks (see the insets in Fig. 2).

**Figure 3:**
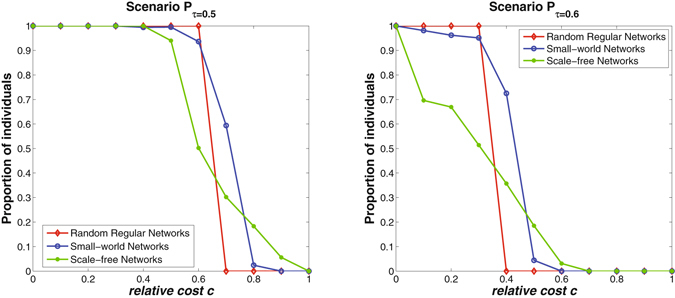
The pure effects of network structure on individuals’ vaccinating strategies. The left figure shows the average proportion of individuals with vaccinating strategy *p*_*i*_ > 0.5; The right figure shows the average proportion of individuals with vaccinating strategy *p*_*i*_ > 0.6. The results are averaged over 50 independent network simulations with size *N* = 5000 and average degree *k* = 4. As the relative cost *c* increases, there is a phase transition from 100 percent to 0 for all types of networks. It can be observed that the proportion of individuals with high vaccinating willingness (i.e., *p*_*i*_(∞) > 0.5) decreases more abruptly in random regular networks than in small-world and scale-free networks.

Based on the estimated infection force *β* for each type of complex networks, simulations are conducted to evaluate the pure effects of network structure on individuals’ vaccinating behaviors. Instead of the average vaccinating probability of all individuals (used in Fig. 1), the proportion of individuals with vaccinating strategies *p*_*i*_(∞) > 0.5 (denoted as *P*_*τ* = 0.5_) and *p*_*i*_(∞) > 0.6 (denoted as *P*_*τ* = 0.6_) are counted to reflect the diversity of individuals willingness to vaccinate in different networks. Figure 3 shows the simulation results on three types of complex networks with respect to different values of relative cost *c*. The results are averaged over 50 independent network simulations with size *N* = 5000 and average degree *k* = 4. It can be observed that as the relative cost *c* increases, both *P*_*τ* = 0.5_ and *P*_*τ* = 0.6_ have a phase transition from 100 percent to 0 for all three types of networks. The proportion of individuals with high vaccinating willingness decreases more abruptly in random regular networks than in small-world and scale-free networks. The main reason is that individuals in random regular networks have more homogeneous degree distributions than that in small-world and scale-free networks. In other words, individuals have similar local environment in random regular networks, which makes it more easily for individuals to perform similar vaccinating strategies through interacting with each other. For example, in a random regular network, when the relative cost reaches a critical value (i.e., a value between 0.6 and 0.7 for the scenario *P*_*τ* = 0.5_ in Fig. 3), almost all individuals will rapidly change their vaccinating willingness. Similar observation can also be found for the scenario *P*_*τ* = 0.6_.

### Network degree and individual vaccinating strategy

**Figure 4:**
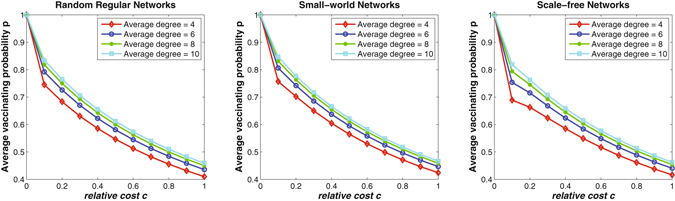
The effects of average degree on individuals’ vaccinating strategies for random regular networks, small-world networks, and scale-free networks. The results are averaged over 50 independent network simulations with size *N* = 5000. As the average degree *k* increases, the vaccinating probability of all individuals also increases for different values of relative cost on all three types of networks.

**Figure 5:**
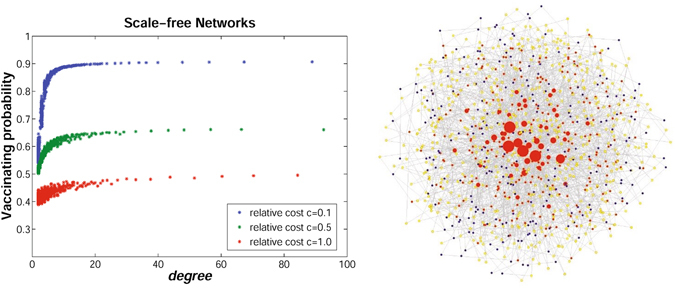
The effects of degree heterogeneity on individuals’ vaccinating strategies with *β* = 0.55 in scale-free networks. The left figure shows the relationship between individuals’ degrees and their vaccinating strategies on scale-free networks. The blue points show individuals’ vaccinating probabilities when the relative cost *c* = 0.1; the green points show individuals’ vaccinating probabilities when the relative cost *c* = 0.5; and the red points show individuals’ vaccinating probabilities when the relative cost *c* = 1.0. The right figure shows the snapshot of individuals’ degrees and vaccinating strategies in a scale-free network when *c* = 0.1. The higher the node degree, the larger the node size. The red nodes represents individuals with high vaccinate probability; the blue nodes represents individuals with middle vaccinate probability; the yellow nodes represents individuals with low vaccinate probability.

Since network structure may highly affect individuals’ vaccinating behaviors (see Fig. 3), simulations are then carried out to study the effects of average degree on individuals’ vaccinating strategies. Figure 4 demonstrates the simulation results on random regular networks, small-world networks, and scale-free networks. It can be observed that as the average degree increases, the average vaccinating probability of all individuals increase on all the three types of networks. This is reasonable because the higher the average degree is, the larger the interacting group each individual belongs to. Individuals in a large group are more likely to be infected that those in a small group. Taking a step forward, the relationships between individuals’ vaccinating strategies and their degree diversity are further investigated. Simulations are conducted only on scale-free networks, which have the most heterogeneous degree among the three types of networks. Without loss of generality, the network size is set to be *N* = 1000, and the average degree is set to be *k* = 4. The left figure in Fig. 5 depicts the scatter diagram of individual degree and the final vaccinating strategy with respect to three scenarios, where the relative cost *c* is set to be 0.1, 0.5, and 1.0, respectively. It can be observed that for small relative cost (e.g., *c* = 0.1), individuals’ final vaccinating probabilities vary widely. While as the relative cost increases (e.g., *c* = 1.0), there is no significant difference between individuals’ vaccinating strategies. This is because a larger relative cost *c* will dominate the perceived risk of infection *r*(*p*) during individuals’ decision-making procedure (see Equation (11) in Methods section). Further, it can also be observed from Fig. 5 that individuals with high degree have higher willingness to vaccinate than those with low degree in scale-free networks. The reason is that the risk of infection for individuals with high degree is usually larger than those with lower degree for they are more likely to contact with infectious individuals. The right figure in Fig. 5 shows the snapshot of the final vaccinating probabilities in a scale-free network when relative cost *c* = 0.1. The higher the node degree, the larger the node size. The red nodes represents individuals with high vaccinating probability (i.e., *p*_*i*_(∞) > 0.85); the blue nodes represents individuals with middle vaccinating probability (i.e., 0.7 < *p*_*i*_(∞) < 0.85); and the yellow nodes represents individuals with low vaccinating probability (i.e., *p*_*i*_(∞) < 0.7).

### The effects of “neighbors of neighbors”

**Figure 6:**
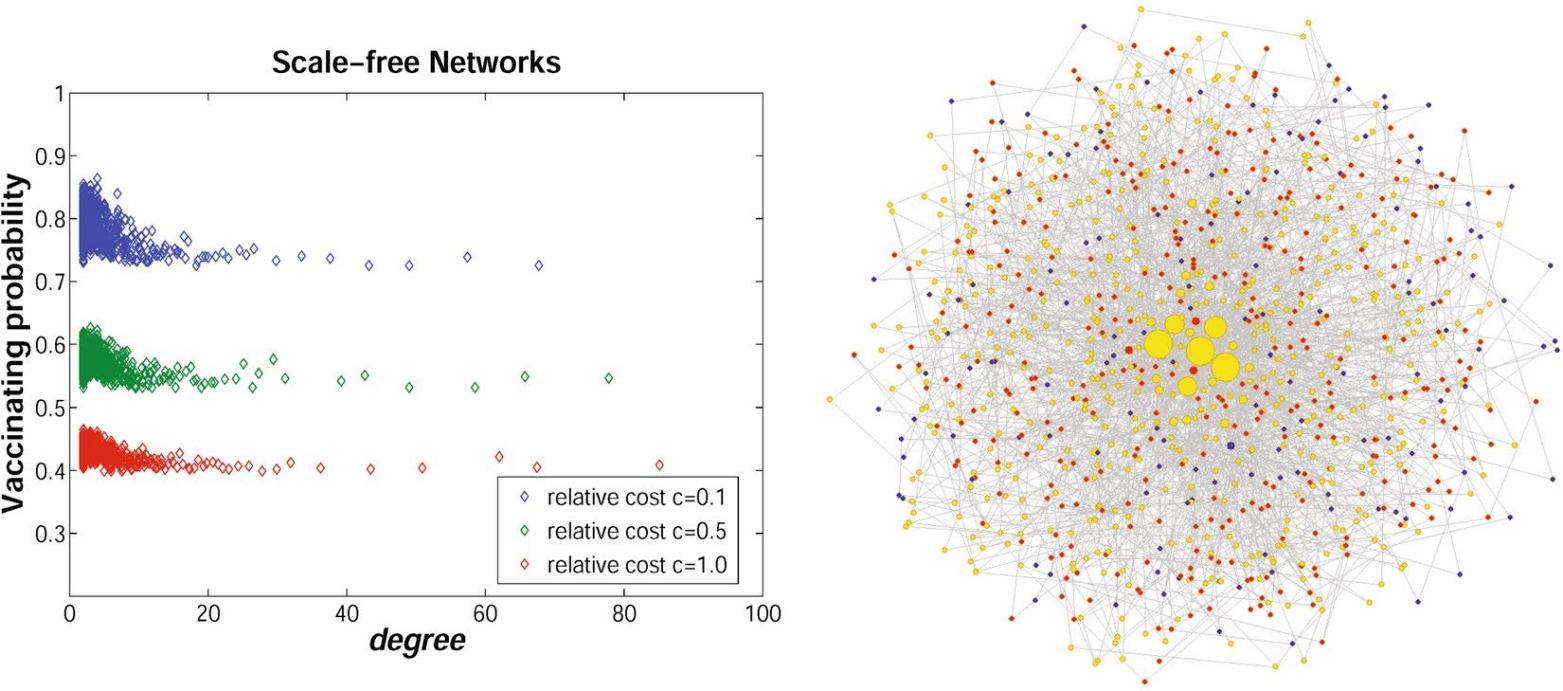
The effects of degree heterogeneity on individuals’ vaccinating strategies by taking into consideration the “neighbors of neighbors”. The left figure shows the relationship between individuals’ degrees and their vaccinating strategies on scale-free networks. The blue points show individuals’ vaccinating probabilities when the relative cost *c* = 0.1; the green points show individuals’ vaccinating probabilities when the relative cost *c* = 0.5; and the red points show individuals’ vaccinating probabilities when the relative cost *c* = 1.0. The right figure shows the snapshot of individuals’ degrees and vaccinating strategies in a scale-free network when *c* = 0.1. The higher the node degree, the larger the node size. The red nodes represents individuals with high vaccinate probability; the blue nodes represents individuals with middle vaccinate probability; the yellow nodes represents individuals with low vaccinate probability.

Till now, simulations are carried out by assuming that individuals have only information about their direct neighbors in locally-mixed complex networks. It is still unclear how individuals’ expanded awareness affects the evolutionary dynamics of their vaccinating behaviors, where each individual has complete information about his/her “neighbors of neighbors”. Similarly, simulations are conducted only on scale-free networks with size *N* = 1000 and average degree *k* = 4. The left figure in Fig. 6 shows the scatter diagram of individual degree and final vaccinating probability for *c* = 0.1, 0.5, and 1.0, respectively. It can be observed that contrary to the results in Fig. 5, individuals with high degree have relatively lower willingness to vaccinate (see yellow nodes in the right figure of Fig. 6). Moreover, except for certain individuals with low degree, there is no significant differences between nodes with high and middle degree in terms of the final vaccinating probability. This is because in scale-free networks, most individuals will belong to a large group centered by a small number of hub nodes through their “neighbors of neighbors”. In this case, individuals with lower degree may overestimate the risk of infection, and then take vaccine with high probability. Therefore, the hub nodes are more likely to benefit from vaccinating strategies of peripheral nodes. The two seemingly contradictory observations in Figs 5 and 6 are mainly determined by the individual’s cognitive ability about the epidemic dynamics, as well as their social relationships.

## Discussion

Since in reality, people usually interact with each other in groups, in this paper, we have studied self-organizing voluntary vaccination on complex networks by assuming locally-mixed local environment. Under this assumption, individuals form a “network of networks” through their social contact relationships. In a homogeneously mixed neighborhood, individuals can make *rational* vaccinating decisions based on their neighbors’ strategies, which is different from pairwise interactions (e.g., imitation) in most existing studies^40, 41, 43–45^. Moreover, individuals in this paper adopt *mixed strategy* rather than pure strategy, which makes it possible to investigate the vaccinating willingness for certain types of individuals. For example, it has been found that the proportion of individuals with high vaccinating willingness deceases more slowly in heterogeneous network structure (e.g., scale-free networks) than that in regular network structure (e.g., square lattices). Meanwhile, individuals with high degree are more likely to take vaccine if they consider only vaccinating strategies of their direct neighbors. However, if individuals have complete information about their “neighbors of neighbors”, individuals with high degree will benefit from vaccinating behaviors of other individuals due to the effects of herd immunity.

Previous studies have shown that targeted immunization against nodes with large degree in scale-free networks can considerably lower the vulnerability of a network to epidemic outbreaks over random immunization^57–59^. While the findings in this paper reveal that individuals with large degree may already have high willingness to vaccinate by assuming that individuals are homogeneously mixed in their local environment. The results are consistent with each other because hub nodes have high risk of infection. From the perspective of disease intervention, it is straightforward to immunize the hub nodes to prevent the epidemic spreading. This is one typical implementation of ring vaccination, which hinders the spread of a disease by vaccinating only those who are most likely to be infected. However, evidence shows that ring vaccination is not always successful especially when infectious cases cannot be rapidly diagnosed^60^. From the perspective of vaccination policy-making, it is essential and necessary to design appropriate incentive mechanisms^27^ such that individuals with low vaccinating willingness can be motivated to voluntarily take vaccine. To achieve this, both individuals’ social roles in their contact networks and their awareness about other individuals should be carefully considered.

In recent years, many researchers have explored the dynamics of voluntary vaccination by simulating both the epidemic spreading and human vaccinating behaviors at the same time^5–7, 37, 38^. The main challenges in these studies is how to truly reflect the interaction between disease transmission and human responses. Small changes in the initial parameters may result in large differences in final conclusions. Usually, it is assumed that individuals make vaccinating decisions only when the presence of symptoms in their neighbors^37^. However, this is more or less unrealistic because humans are not so short-sighted in most cases. Like the proposed models in this paper, individuals can get information from their neighbors, or even their “neighbors of neighbors” to make vaccinating decisions by weighing the costs of infection and vaccination.

On the other hand, many researchers have also studied the dynamics of human vaccinating behaviors for seasonal epidemics^40, 41, 43^. In these studies, the vaccinating decisions are made based on social learning from their successful neighbors and their past experiences before the seasonal epidemic begins. By doing so, the dynamics of epidemic spreading and human vaccinating behaviors can be naturally separated. However, because the spread of a disease is a highly stochastic process, a few past experiences are not well suited as the primary basis for future decision-making. Moreover, in most cases, when an infectious disease reemerges, its nature will also change. For example, outbreaks of seasonal influenza viruses are often accompanied by mutations. As a result, past vaccination and infection experiences do not apply to the present epidemic situation. In view of this, the model and methods proposed in this paper can deduce individuals vaccinating willingness through their self-organization behaviors before an disease spreads. The simulation results can even help decision-makers develop appropriate vaccination strategies and incentive mechanisms for emerging infectious diseases based on individuals social contact networks.

Although the SIR model has been extensively adopted to characterize the epidemic dynamics of infectious diseases like smallpox and measles^17, 18, 24, 25^, more complicated analytic and simulation methods, as well as disease transmission models, are needed to characterize the interplay between diseases epidemics and human vaccinating behaviors. However, as with any model, the proposed model has made several simplifying assumptions. For example, it is assumed that individuals make decisions based on strategies of their direct neighbors or “neighbors of neighbors”. In reality, individuals could also get information from various social medias. To address this limitation, the impacts of public information on individuals’ self-organizing behaviors should be further studied. Moreover, it is also assumed that all individuals are rational to make vaccinating decisions. However, people often tend to exaggerate the negative effects of vaccination failure and complications. Therefore, it would be essential to study individuals self-organizing behaviors with bounded rationality^61^, as well as the effects of their memory and adaptability for past vaccinating events^27^. Finally, all findings in this paper are obtained by simulating individuals’ behaviors in synthetic networks. In the future, there remains a need to understand the interplay between disease prevalence and individual vaccinating behavior based on real-world social contact networks^12, 62^. Such real-world networks can be estimated from human mobile data, census data, and other data acquisition techniques.

## Methods

### Epidemic modeling and simulations on networks

In epidemiology, the classical Susceptible-Infected-Recovered (SIR) model has been widely studied to simulate the transmission dynamics of infectious diseases like smallpox and measles^17, 18, 24, 25, 63^. In the SIR model, the population is divided into three states: susceptible (*S*), infected (*I*), and recovered (*R*). Denote *S*, *I*, and *R* as the proportion of susceptible, infected, and recovered individuals, respectively. Then, we have *S* + *I* + *R* = 1. Then, for a well-mixed population, the evolution of population states can be formulated as a deterministic ordinary differential equation:

1 $$\frac{dS}{dt}=-\beta SI,$$

2 $$\frac{dI}{dt}=\beta SI-\gamma I,$$

3 $$\frac{dR}{dt}=\gamma I,$$

where *β* is the number of effective contacts per susceptible individual per day that are sufficient to spread the disease, and *γ* is the recovery rate. There exists a threshold that governs the time evolution of the epidemic process, that is, the so-called *basic reproduction number* (i.e., *R*_0_ = *β*/*γ*). Epidemiologically, *R*_0_ represents the number of secondary infections caused by a single primary infection in a completely susceptible population. When *R*_0_ < 1, each infectious person will infect fewer than one person before recovering, so the outbreak will gradually disappear. When *R*_0_ > 1, each infectious person will infect more than one person, so the epidemic will spread. According to the notion of *herd immunity*, when a critical proportion of the population (i.e., *p*_*c*_) becomes to be vaccinated, the disease may no longer persist in the population. Mathematically, we have *β*(1 − *p*_*c*_)/*γ* = 1, that is, *p*_*c*_ = 1 − 1/*R*_0_. The critical value for herd immunity may cause a public-goods dilemma among self-interested individuals for disease eradication.

Here, we use Gillespie algorithm to simulate the process of an epidemic in both well-mixed and structured population^64^. Like the work of Fu *et al*.^40^, we fix the recovery rate at *γ* = 1/3 day^−1^. We carry out two kinds of experiments: first, we evaluate the effects of infection force on individuals’ self-organizing vaccinating behaviors by setting different values of *β* in various types of networks (i.e., regular, random regular, small-world and scale-free networks). Second, we identify only the effects of network structure on the dynamics of voluntary vaccination by calibrating the values of *β* for different networks. The value of *β* for each network is chosen by simulation such that the final epidemic size (≈ 0.893) is that of the well-mixed population with *R*_0_ = 2.5. Specifically, we assume that the infection probability of a susceptible individual *i* is proportional to the number of infected individuals *I*_*N*(*i*)_ in his/her neighborhood *N*(*i*), that is, the infection rate from susceptible to infected for individual *i* is *βI*_*N*(*i*)_^63^.

### Individuals’ rational strategy in well-mixed environment

We first derive the risk of infection for individuals in a well-mixed population when some of them get protected through vaccination. Suppose the proportion of vaccinated individuals in the population is *p*. Then, we have *S* + *I* + *R* = (1 − *p*). Let $\bar{S}=S\mathrm{/(1}-p)$, $\bar{I}=I\mathrm{/(1}-p)$, and $\bar{R}=R\mathrm{/(1}-p)$, the evolution of population states becomes:

4 $$\frac{d\bar{S}}{dt}=-\beta \bar{S}\bar{I}\mathrm{(1}-p),$$

5 $$\frac{d\bar{I}}{dt}=\beta \bar{S}\bar{I}\mathrm{(1}-p)-\gamma \bar{I},$$

6 $$\frac{d\bar{R}}{dt}=\gamma \bar{I}\mathrm{.}$$

Equation 6 above is superfluous because $\bar{S}+\bar{I}+\bar{R}=1$. Therefore, the remaining equations can be written as:

7 $$\frac{d\bar{S}}{d\tau }=-{R}_{0}\bar{S}\bar{I}\mathrm{(1}-p),$$

8 $$\frac{d\bar{I}}{d\tau }={R}_{0}\bar{S}\bar{I}\mathrm{(1}-p)-\bar{I},$$

where *τ* = *t*/*γ* is time measure in units of the mean infectious period. Dividing the two equations, we have:

9 $$\frac{d\bar{I}}{d\bar{S}}=\frac{1}{\bar{S}{R}_{0}\mathrm{(1}-p)}-1.$$

The evolutionary dynamics will converge to a steady state when $\bar{S}(\infty )\cdot {R}_{0}\mathrm{(1}-p)=1$, which means that the final proportion of uninfected individuals in the original susceptible individuals is $\bar{S}(\infty )=\mathrm{1/(}{R}_{0}\mathrm{(1}-p))$. In other words, there will be $1-\bar{S}(\infty )$ (i.e., 1 − 1/(*R*_0_(1 − *p*))) proportion of susceptible individuals will be infected, and finally recovered. Therefore, in the well-mixed population, when a proportion *p* of the population is vaccinated, the risk of infection for a susceptible individual should be:

10 $$r(p)=1-\frac{1}{{R}_{0}\mathrm{(1}-p)}\mathrm{.}$$

In this paper, we assume that individuals are rational, and adopt a mixed vaccinating strategy (i.e., the probability of vaccination), rather than a pure strategy (i.e., whether or not to vaccine), to reflect their willingness to vaccinate. We study the vaccinating dynamics from an evolutionary perspective. Specifically, the simulation proceeds a number of rounds. At round *t*, an individual *i* can strategically adjust his/her vaccinating strategy *p*_*i*_(*t*) by weighing the costs of vaccination and infection based on other individuals strategies at previous round *t* − 1. Suppose all other individuals do not change their vaccinating decisions, at round *t*, the mixed strategy of individual *i* (i.e., ${p}_{i}^{\ast }(t)$) can be calculated by equalizing the cost of perceived infection and the cost of vaccination. Mathematically, we have $r(p\mathrm{)(1}-{p}_{i}^{\ast }(t))\ast {C}_{I}={p}_{i}^{\ast }(t)\ast {C}_{V}$, where *p* represents the average vaccinating strategy of all individuals at *t* − 1, *C*_*V*_ and *C*_*I*_ represent the cost of vaccination and infection. Therefore, individual *i*’s vaccinating strategy at equilibrium can be calculated as:

11 $${p}_{i}^{\ast }(t)=\frac{r(p)}{r(p)+c},$$

where *c* = *C*_*V*_/*C*_*I*_ is the relative cost of vaccination over infection.

### Self-organizing behaviors in locally-mixed networks

**Figure 7:**
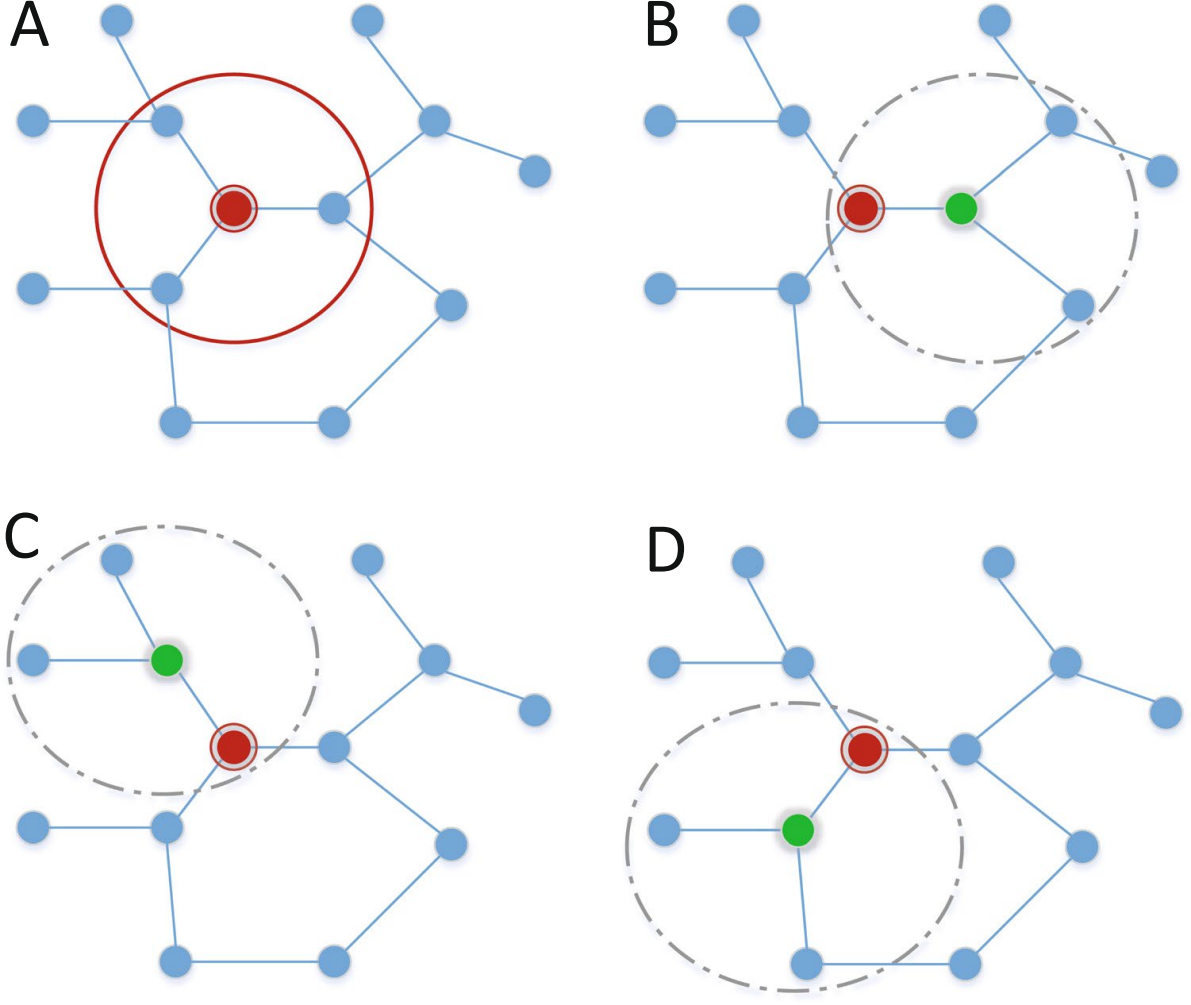
Locally-mixed groups on networks. The focal individual (large red disk) has complete information about his/her three neighbors. Meanwhile, the vaccinating strategy of the focal individual will affect the other individuals’ strategies in gourps B-D certered on his/her neighbors. Individuals in each group (A-D) are assumed to be homogeneously mixed with each other.

Since it is almost impossible to derive explicit equations for epidemic spreading in structured populations, stochastic simulations of individuals self-organizing behaviors are used in this work. To simulate individuals’ group activities, it is assumed that all individuals form a “network of networks” through their social contact relationships, each of which together with his/her neighbors form a locally-mixed environment. As shown in Fig. 7, the focal individual (large red disk) forms a locally-mixed group (A) with his/her three neighbors. At each round *t*, the focal individual in group A adjusts his/her vaccinating strategy based on other individuals’ strategies at previous round *t* − 1. Meanwhile, the vaccinating strategy of the focal individual will also affect the vaccinating decisions of his/her neighbors in groups B-D. Specifically, the strategy is updated as follows:

12 $${p}_{i}(t)={p}_{i}(t-\mathrm{1)}+\lambda ({p}_{i}^{\ast }(t)-{p}_{i}(t-\mathrm{1)),}$$

where *λ* represents the step size for updating. The effects of different step sizes will be evaluated in our experiments.

The simulations in this paper capture individuals’ strategic interactions in the following ways. Initially, all individuals’ vaccinating strategies are randomly assigned from [0,1]. Then, it will be adjusted by interacting with other individuals in his/her local environment based on Equation (12). The process will terminate when all individuals’ strategies converge to a steady state. In this paper, to evaluate the effects of different network structures on individuals’ self-organizing vaccinating behaviors, simulations are conducted on three types of complex networks, that is, random regular networks, small-world networks, and scale-free networks. During each simulation, the variations of individual vaccinating probabilities *p*_*i*_(*t*) are recorded. The average vaccinating probability of all individuals (*p* = ∑_*i*_ *p*_*i*_(∞)) are then used as an indicator to reflect the evolutionary dynamics of individuals’ vaccinating behaviors. Here, *p*_*i*_(∞) represents *i*’s vaccinating probability at the steady state of the evolutionary dynamics. Finally, the average vaccinating probability *p* are averaged over 50 independent network simulations.

### Decision-making through “neighbors of neighbors”

Except for interacting with direct neighbors in the locally-mixed group, an individual can also make vaccinating decisions based on his/her “neighbors of neighbors”. For example, the focal individual of group A in Fig. 7 may further have information about individuals in groups B, C, and D, where group members are homogeneously mixed with each other. At each round, the focal individual will weigh the costs of vaccination and infection, and calculate the optimal vaccinating strategy in each group based on Equation (11). Then, the maximum vaccinating probability among all groups is chosen to update his/her vaccinating strategy based on Equation (12). The consideration of “neighbors of neighbors” reflects individuals expanded awareness about the epidemic dynamics, as well as other individuals’ behaviors. In this paper, the effects of “neighbors of neighbors” on individuals’ vaccinating decision-making are evaluated by comparing with the situation where only the focal group is considered.
